# Complete genome sequence of *Hirschia baltica* type strain (IFAM 1418^T^)

**DOI:** 10.4056/sigs.2205004

**Published:** 2011-12-23

**Authors:** Olga Chertkov, Pamela J.B. Brown, David T. Kysela, Miguel A. de Pedro, Susan Lucas, Alex Copeland, Alla Lapidus, Tijana Glavina Del Rio, Hope Tice, David Bruce, Lynne Goodwin, Sam Pitluck, John C. Detter, Cliff Han, Frank Larimer, Yun-juan Chang, Cynthia D. Jeffries, Miriam Land, Loren Hauser, Nikos C. Kyrpides, Natalia Ivanova, Galina Ovchinnikova, Brian J. Tindall, Markus Göker, Hans-Peter Klenk, Yves V. Brun

**Affiliations:** 1 DOE Joint Genome Institute, Walnut Creek, California, USA; 2 Los Alamos National Laboratory, Bioscience Division, Los Alamos, New Mexico, USA; 3 Indiana University, Bloomington, Indiana, USA; 4 Universidad Autonoma de Madrid, Campus de Cantoblanco, Madrid, Spain; 5 Oak Ridge National Laboratory, Oak Ridge, Tennessee, USA; 6 DSMZ – German Collection of Microorganisms and Cell Cultures, Braunschweig, Germany

**Keywords:** aerobic, chemoheterotrophic, mesophile, Gram-negative, motile, budding, stalk-forming, *Hyphomonadaceae*, *Alphaproteobacteria*, CSP 2008

## Abstract

The family *Hyphomonadaceae* within the *Alphaproteobacteria* is largely comprised of bacteria isolated from marine environments with striking morphologies and an unusual mode of cell growth. Here, we report the complete genome sequence *Hirschia baltica*, which is only the second a member of the *Hyphomonadaceae* with a published genome sequence. *H. baltica* is of special interest because it has a dimorphic life cycle and is a stalked, budding bacterium. The 3,455,622 bp long chromosome and 84,492 bp plasmid with a total of 3,222 protein-coding and 44 RNA genes were sequenced as part of the DOE Joint Genome Institute Program CSP 2008.

## Introduction

Strain IFAM 1418^T^ (= ATCC 49814 = DSM 5838 = KCTC 12471) is the type strain of the species *Hirschia baltica*, which is the type species of the genus *Hirschia.* The genus name *Hirschia* Baker 1896 has been applied to a member of the *Asteraceae*, although the type and only species, *Hirschia anthemidifolia* has also been placed in the genus *Iphiona* [1]. The genus *Hirschia* was named in honor of Peter Hirsch, a German microbiologist and expert on budding and hyphal bacteria. The species epithet refers to the Neo-Latin *baltica*, pertaining to the Baltic Sea [2]. IFAM 1418^T^ was isolated in 1982 along with two additional stalk-forming isolates belonging to the same species, IFAM 1408 and IFAM 1415, from a sample of surface water taken from a boat landing in the Kiel Fjord [2].

*H. baltica* reproduces by budding from the tip of a stalk and has a dimorphic life-cycle similar to that described in bacteria belonging to the closely related genus *Hyphomonas* [2]. Newborn swarmer cells are motile by means of a polar flagellum. Swarmer cells differentiate into stalked sessile cells by ejecting the flagellum and synthesizing a stalk at the same pole. The sessile cells reproduce by budding such that motile daughter cells are formed at and released from the tip of the stalk. Here we present a summary classification and a set of features for *H. baltica* IFAM 1418^T^, together with the description of the complete genomic sequencing and annotation.

## Classification and features

A representative genomic 16S rRNA sequence of *H. baltica* was compared using NCBI BLAST [3] under default settings (e.g., considering only the high-scoring segment pairs (HSPs) from the best 250 hits) with the most recent release of the Greengenes database [4] and the relative frequencies of taxa and keywords (reduced to their stem [5]) were determined, weighted by BLAST scores. The most frequently occurring genera were *Hyphomonas* (37.8%), *Maricaulis* (29.5%), *Hirschia* (14.3%), *Caulobacter* (7.4%) and *Woodsholea* (3.6%) (74 hits in total). Regarding the four hits to sequences from members of the *Hirschia* species, the average identity within HSPs was 99.3%, whereas the average coverage by HSPs was 98.8%. Among all other species, the one yielding the highest score was *Hyphomonas johnsonii* (NR_024938), which corresponded to an identity of 93.1% and an HSP coverage of 59.6%. (Note that the Greengenes database uses the INSDC (= EMBL/NCBI/DDBJ) annotation, which is not an authoritative source for nomenclature or classification.) The highest-scoring environmental sequence was FR684125 ('effect ocean acidification upon microbial prokaryotes marine biome fjord coastal water clone 16 07 04A09'), which showed an identity of 89.4% and an HSP coverage of 99.0%. The most frequently occurring keywords within the labels of environmental samples which yielded hits were 'marin' (2.2%), 'microbi' (2.1%), 'water' (2.0%), 'biofilm' (1.9%) and 'sea' (1.6%) (176 hits in total). Environmental samples which yielded hits of a higher score than the highest scoring species were not found. These keywords reflect very well some the ecological and physiological properties reported for strain IFAM 1418^T^ in the original description [2].

**Figure 1:**
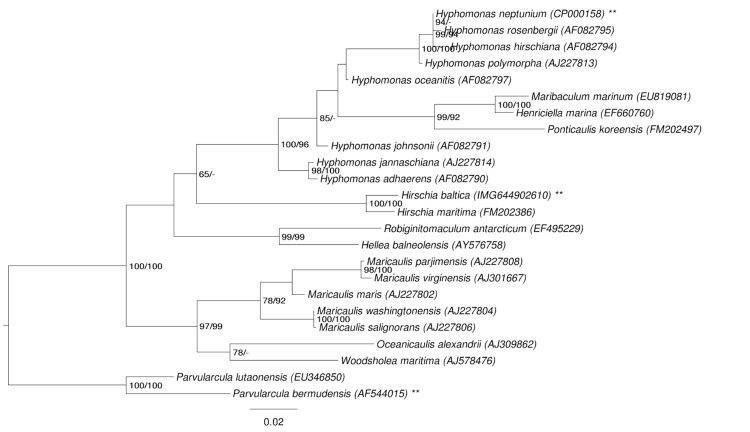
Phylogenetic tree highlighting the position of *H. baltica* relative to the type strains of the other species within the family *Hyphomonadaceae*. The tree was inferred from 1,332 aligned characters [6,7] of the 16S rRNA gene sequence under the maximum likelihood (ML) criterion [8] and rooted with the neighboring family *Parvularculaceae*. The branches are scaled in terms of the expected number of substitutions per site. Numbers adjacent to the branches are support values from 450 ML bootstrap replicates [9] (left) and from 1,000 maximum parsimony (MP) bootstrap replicates [10] (right) if larger than 60%. Lineages with type strain genome sequencing projects registered in GOLD [11] are labeled with one asterisk, those also listed as 'Complete and Published' (as well as the target genome) with two asterisks [12,13].

Figure 1 shows the phylogenetic neighborhood of *H. baltica* in a 16S rRNA based tree. The sequences of the two identical 16S rRNA gene copies in the genome differ by one nucleotide from the previously published 16S rRNA sequence (AJ421782), which contains one ambiguous base call.

**Figure 2:**
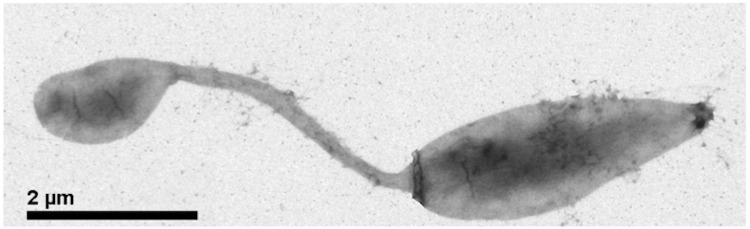
Scanning Electron micrograph of *H. baltica* IFAM 1418^T^

Cells of *H. baltica* strain IFAM 1418^T^ are rod-shaped, elliptical or ovoid with 0.5 – 1.0 by 0.5 – 6.0 µm in size (without hyphae which have a diameter of about 0.2 µm) (Figure 2) [2]. IFAM 1418^T^ cells stain Gram-negative, are motile and strictly aerobic [2]. 1 to 2 hyphae are located polarly and flagellation is monotrichous polar [2]. Cells grow best in artificial sea water with a broad range of NaCl concentrations (Table 1). The strain grows on a broad spectrum of organic compounds as carbon source such as amino acids, organic acids and sugars (Table 1), but not on C_1_ compounds [2]. In contrast to its phylogenetic neighbors *H. baltica* strains do not store PHB [2]. Colonies of strain IFAM 1418^T^ show a yellow pigmentation whereas strain IFAM 1408 colonies are white [2].

**Table 1:** Classification and general features of *H. baltica* according to the MIGS recommendations [14].

**MIGS ID**	**Property**	**Term**	**Evidence code**
	Current classification	Domain *Bacteria*	TAS [15]
		Phylum *Proteobacteria*	TAS [16]
		Class *Alphaproteobacteria*	TAS [17,18]
		Order *Caulobacterales*	TAS [19,20]
		Family *Hyphomonadaceae*	TAS [21]
		Genus *Hirschia*	TAS [2]
		Species *Hirschia baltica*	TAS [2]
		Type strain IFAM 1418	TAS [2]
	Gram stain	negative	TAS [2]
	Cell shape	rod-shaped	TAS [2]
	Motility	motile	TAS [2]
	Sporulation	none	TAS [2]
	Temperature range	mesophile	TAS [2]
	Optimum temperature	22-28°C	TAS [2]
	Salinity	artificial sea water, 0.5 – 8.6% NaCl	TAS [2]
MIGS-22	Oxygen requirement	aerobe	TAS [2]
	Carbon source	various amino acids, organic acids and sugars	TAS [2]
	Energy metabolism	chemoheterotroph	TAS [2]
MIGS-6	Habitat	brackish water	TAS [2]
MIGS-15	Biotic relationship	free living	TAS [2]
MIGS-14	Pathogenicity	none	NAS
	Biosafety level	1	TAS [22]
	Isolation	surface water samples	TAS [2]
MIGS-4	Geographic location	Baltic Sea, Kiel Fjord	TAS [2]
MIGS-5	Sample collection time	October 1982	NAS
MIGS-4.1	Latitude	approximately 54.5	NAS
MIGS-4.2	Longitude	approximately 10.2	NAS
MIGS-4.3	Depth	5 cm	TAS [2]
MIGS-4.4	Altitude	sea level	NAS

Evidence codes - TAS: Traceable Author Statement (i.e., a direct report exists in the literature); NAS: Non-traceable Author Statement (i.e., not directly observed for the living, isolated sample, but based on a generally accepted property for the species, or anecdotal evidence). These evidence codes are from of the Gene Ontology project [23].

### Chemotaxonomy

Cell walls of *H. baltica* contain meso-diamonopimelic acid [2]. The main quinone component is Q_10_ [2,24]. In the original publications by Schlesner *et al.* [2] and Sittig and Hirsch [24] the fatty acids were separated into non-hydroxy fatty acids and hydroxy-fatty acids. Main cellular non-hydroxy fatty acids are C_18:1 Δ11_ (48.8%), C_16:0_ (22.1%), C_18:2 Δ5,11_ (9.5%), C_16:1 Δ5_ (4.6%), and C_14:1 Δ7_ (3.2%). Among the hydroxylated fatty acids IFAM 1418^T^ 3-OH fatty acids with C_14:1 3-OH_ (79%) and C_12:0 3-OH_ (15.8%) predominate, which are useful for the discrimination for *H. baltica* from strains of *Hyphomicrobium* and *Hyphomonas* (Figure 1). Kang and Lee [25] were unable to confirm the presence of these hydoxylated fatty acids in *H. baltica*. There are two reports [2,24] on the lipid composition of *H. baltica* and one on the lipids of *H. maritima* [25]. While all confirm the presence of a single phospholipid, phosphatidylglycerol in members of this genus, it is unclear whether these studies were limited to the detection of only phospholipids. There is evidence that *H. baltica* contains five major lipids, of which one is phosphatidlyglycerol (BJ Tindall, unpublished). The remaining four lipids are not phosphate positive, but their R_f_ values and staining behavior indicate similarities to a number of the unusual lipids reported from *Hyphomonas jannaschiana* [26,27].

## Genome sequencing and annotation

### Genome project history

This organism was selected for sequencing as a part of the DOE Joint Genome Institute Program CSP 2008. The genome project is deposited in the Genomes OnLine Database [11] and the complete genome sequence is deposited in GenBank. Sequencing, finishing and annotation were performed by the DOE Joint Genome Institute (JGI). A summary of the project information is shown in Table 2.

**Table 2:** Genome sequencing project information

**MIGS ID**	**Property**	**Term**
MIGS-31	Finishing quality	Finished
MIGS-28	Libraries used	Three genomic libraries: one 454 pyrosequence standard library, one 454 PE library (24 kb insert size), one Illumina library
MIGS-29	Sequencing platforms	454 GS FLX Titanium, Illumina GAii
MIGS-31.2	Sequencing coverage	116.6 × Illumina; 34.0 x pyrosequence
MIGS-30	Assemblers	Newbler version 2.0.00.20, Velvet, phrap
MIGS-32	Gene calling method	Critica complemented with the output of Glimmer
	INSDC ID	CP001678 (chromosome) CP001679 (plasmid pHbal01)
	GenBank Date of Release	July 19, 2009
	GOLD ID	Gc01064
	NCBI project ID	33191
	Database: IMG	644736375
MIGS-13	Source material identifier	ATCC 49814
	Project relevance	Bioremediation, Biotechnology

### Strain history

The history of strain IFAM 1418^T^ starts with H. Schlesner who independently deposited the strain in two culture collections, ATCC and DSMZ (in 1989).

### Growth conditions and DNA isolation

The culture of strain IFAM 1418^T^ used to prepare genomic DNA (gDNA) for sequencing was obtained directly from the ATCC. ATCC 49814 was grown in Hirschia medium (ATCC medium 1883) at 30°C with shaking.

### Genome sequencing and assembly

The genome was sequenced using a combination of Illumina and 454 sequencing platforms. All general aspects of library construction and sequencing can be found at the JGI website [28]. Pyrosequencing reads were assembled using the Newbler assembler (Roche). The initial Newbler assembly consisting of nine contigs in two scaffolds was converted into a phrap [29] assembly by making fake reads from the consensus, to collect the read pairs in the 454 paired end library. Illumina GAii sequencing data (50 Mb) were assembled with Velvet [30] and the consensus sequences were shredded into 1.5 kb overlapped fake reads and assembled together with the 454 data. The 454 draft assembly was based on 128.0 Mb 454 draft data and all of the 454 paired end data. Newbler parameters were -consed -a 50 - l350 -g -m -ml 20. The Phred/Phrap/Consed software package [29] was used for sequence assembly and quality assessment in the subsequent finishing process. After the shotgun stage, reads were assembled with parallel phrap (High Performance Software, LLC). Possible mis-assemblies were corrected with gapResolution [28], Dupfinisher [31], or sequencing cloned bridging PCR fragments with subcloning. Gaps between contigs were closed by editing in Consed, by PCR and by Bubble PCR primer walks (J.-F. Chang, unpublished). A total of 55 additional reactions were necessary to close gaps and to raise the quality of the finished sequence. Illumina reads were also used to correct potential base errors and increase consensus quality using a software Polisher developed at JGI [28,32]. The error rate of the completed genome sequence is less than 1 in 100,000. Together, the combination of the Illumina and 454 sequencing platforms provided 150.6 × coverage of the genome. The final assembly contained 333,683 pyrosequence and 12,860,398 Illumina reads.

### Genome annotation

Genes were identified using Prodigal [33] as part of the Oak Ridge National Laboratory genome annotation pipeline, followed by a round of manual curation using the JGI GenePRIMP pipeline [34]. The predicted CDSs were translated and used to search the National Center for Biotechnology Information (NCBI) non-redundant database, UniProt, TIGRFam, Pfam, PRIAM, KEGG, COG, and InterPro databases. These data sources were combined to assert a product description for each predicted protein. Non-coding genes and miscellaneous features were predicted using tRNAscan-SE [35], RNAMMer [36], Rfam [37], TMHMM [38], and signalP [39].

## Genome properties

**Figure 3:**
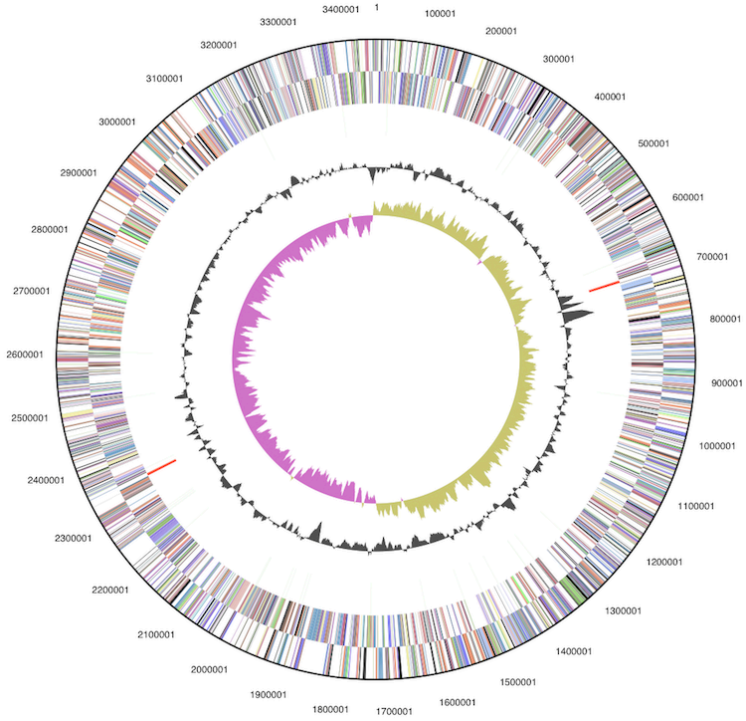
Graphical circular map of the chromosome (plasmid not shown). From outside to the center: Genes on forward strand (color by COG categories), Genes on reverse strand (color by COG categories), RNA genes (tRNAs green, rRNAs red, other RNAs black), GC content, GC skew.

The genome consists of a 3,455,622 bp long chromosome with a 45% G+C content and a 84,492 bp long plasmid with a 44% G+C content (Table 3 and Figure 3). Of the 3,266 genes predicted, 3,222 were protein-coding genes, and 44 RNAs; 35 pseudogenes were also identified. The majority of the protein-coding genes (71.6%) were assigned a putative function while the remaining ones were annotated as hypothetical proteins. The distribution of genes into COGs functional categories is presented in Table 4.

**Table 3:** Genome Statistics

**Attribute**	**Value**	**% of Total**
Genome size (bp)	3,540,114	100.00%
DNA coding region (bp)	3,172,944	89.63%
DNA G+C content (bp)	1,599,770	45.19%
Number of replicons	2	
Extrachromosomal elements	1	
Total genes	3,266	100.00%
RNA genes	44	1.35%
rRNA operons	2	
Protein-coding genes	3,222	98.65%
Pseudo genes	35	1.07%
Genes with function prediction	2,337	71.65%
Genes in paralog clusters	223	6.83%
Genes assigned to COGs	2,443	74.80%
Genes assigned Pfam domains	2,551	78.11%
Genes with signal peptides	797	24.40%
Genes with transmembrane helices	759	23.24%
CRISPR repeats	not reported	

**Table 4:** Number of genes associated with the general COG functional categories

**Code**	**value**	**%age**	**Description**
J	158	5.9	Translation, ribosomal structure and biogenesis
A	0	0.0	RNA processing and modification
K	167	6.2	Transcription
L	106	3.9	Replication, recombination and repair
B	1	0.0	Chromatin structure and dynamics
D	28	1.0	Cell cycle control, cell division, chromosome partitioning
Y	0	0.0	Nuclear structure
V	34	1.3	Defense mechanisms
T	140	5.2	Signal transduction mechanisms
M	196	7.3	Cell wall/membrane biogenesis
N	70	2.6	Cell motility
Z	0	0.0	Cytoskeleton
W	0	0.0	Extracellular structures
U	92	3.4	Intracellular trafficking and secretion, and vesicular transport
O	129	4.8	Posttranslational modification, protein turnover, chaperones
C	152	5.7	Energy production and conversion
G	141	5.2	Carbohydrate transport and metabolism
E	204	7.6	Amino acid transport and metabolism
F	55	2.1	Nucleotide transport and metabolism
H	119	4.4	Coenzyme transport and metabolism
I	133	5.0	Lipid transport and metabolism
P	152	5.7	Inorganic ion transport and metabolism
Q	82	3.1	Secondary metabolites biosynthesis, transport and catabolism
R	287	10.7	General function prediction only
S	243	9.0	Function unknown
-	823	25.2	Not in COGs

## Notable features of the *H. baltica* genome sequence

### *H. baltica* genome is enriched in TonB-dependent receptors

TonB-dependent receptors are outer membrane proteins that function in the energy-dependent uptake of macromolecules, including siderophores and vitamins, that are too large to be taken up by passive diffusion. The genome sequences of *C. crescentus* and *H. neptunium* contain large numbers (63 and 43, respectively) of TonB dependent receptors [40,41]. One possibility is that the presence of many TonB-dependent receptors is a common feature among bacteria belonging to the order *Caulobacterales*. Indeed, the annotation of the *H. baltica* genome sequence suggests that there are 46 TonB-dependent receptors. The presence of many TonB-dependent receptors may be part of the adaptations that have allowed the *Caulobacterales* to not only inhabit, but also thrive in low-nutrient environments.

### *H. baltica* genome contains known regulators of the cell cycle

A recent bioinformatic analysis of genes controlling the dimorphic cell cycle within the *Alphaproteobacteria* suggests the circuitry for cell-cycle control is largely conserved [42]. The conservation of 14 key proteins that function in the regulation of the cell cycle in *C. crescentus* was addressed among the *Alphaproteobacteria* with sequenced genomes, including three bacteria belonging to the *Caulobacterales*. All of the regulatory proteins were conserved among the genomes of the *Caulobacterales* with one notable exception: DivJ, a histidine kinase, is absent in the *H. neptunium* genome.

Expanding the analysis to include 8 additional bacteria belonging to the *Caulobacterales*, including *H. baltica*, we find that all 14 regulatory proteins are conserved with the exception of DivJ, which is absent only from the *H. neptunium* and *H. baltica* genomes. The fact that most developmental regulators are conserved in the budding bacteria *H. neptunium* and *H. baltica* as well as the non-budding bacteria belonging to the *Caulobacterales* suggests that regulation of the cell cycle is evolved prior to the separation of the budding and non-budding bacteria in the *Caulobacterales*. The finding that DivJ is absent from *H. neptunium* and *H. baltica* but present in the closely related non-budding *Maricaulis maris* and *Oceanicaulis alexandrii* (Figure 1) is an intriguing observation, although the significance of this finding remains unknown.

### *H. baltica* genome contains genes for holdfast synthesis and attachment

**Figure 4:**
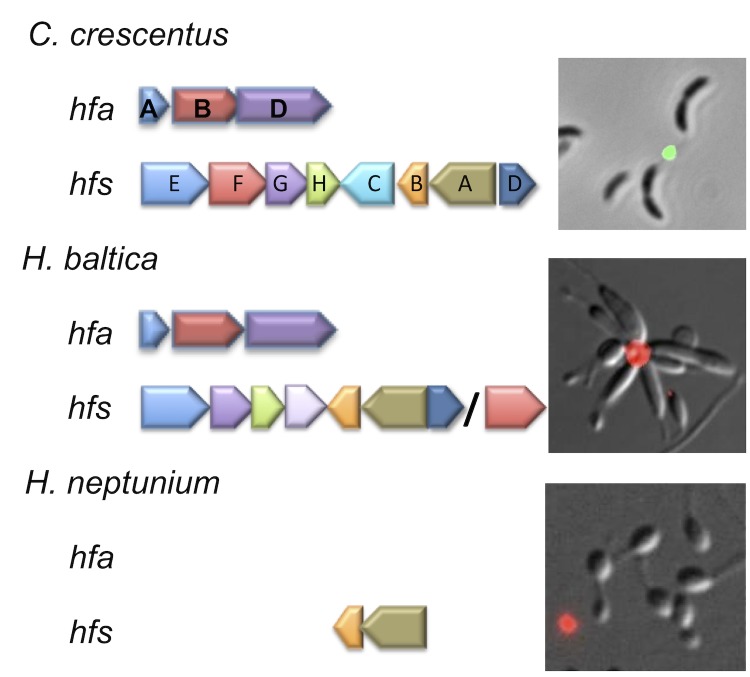
Holdfast anchor (*hfa*) and synthesis (*hfs*) gene cluster conservation is depicted among *C. crescentus*, *H. baltica* and *H. neptunium*. Presence of holdfast genes is correlated to the ability to detect polar holdfast polysaccharide using fluorescent wheat germ agglutinin lectin. The *H. neptunium* genome contains only *hfsAB* and fails to make a polar polysaccharide. In contrast, the *H. baltica* genome contains all essential *hfs* and *hfa* genes and produces a holdfast.

Bacteria belonging to the order *Caulobacterales* are known for the ability to produce a polar polysaccharide, termed holdfast, which mediates strong adhesion to surfaces (For review see [43]). Notably, extracellular polysaccharides from some of the stalked bacteria sequester metals [44,45], a feature that could be used to remediate environments affected by metal toxicity. The genes required for the synthesis [46,47] and anchoring [48] of the holdfast have been identified and characterized in *C. crescentus*. The holdfast synthesis and anchor genes are largely absent from the genome of *H. neptunium*, which does not produce a polar holdfast (Figure 4 and [41]). The genome sequence of *H. baltica* revealed that the genes predicted to be involved in polar holdfast synthesis are present (Figure 4). Furthermore, a holdfast on *H. baltica* cells was readily detected using a fluorescent wheat germ agglutinin lectin using the procedure detailed in [44] (Figure 4). The holdfast of *H. baltica* is found at the cell pole opposite the stalk of the mother cell and is responsible for the formation of star-shaped cell aggregates known as rosettes in cell culture (Figure 4). This finding, coupled with the observation that other species of *Hyphomonas* produce detectable holdfasts [49], suggests the ability to synthesize holdfast is a conserved feature among the *Hyphomonadaceae* family and the loss of the holdfast synthesis and anchoring genes in *H. neptunium* was a recent evolutionary event.

## Concluding Remarks

The completion of the *H. baltica* genome sequence is expected to provide a valuable resource for understanding the biology of stalked budding bacteria with a dimorphic life cycle. Further comparative genomic analyses (especially as more bacteria belonging to the *Caulobacterales* are sequenced) are likely to provide insights regarding the evolution, mechanism, and advantages underlying processes such as budding, and stalk synthesis. Understanding stalk biosynthesis is of particular interest as the ability of these structures to transport nutrients [50,51] could likely be exploited to facilitate the uptake of toxic compounds from contaminated water sources.
